# The Combined Effect of Licorice Extract and Bone Marrow Mesenchymal Stem Cells on Cisplatin-Induced Hepatocellular Damage in Rats

**DOI:** 10.3390/metabo13010094

**Published:** 2023-01-06

**Authors:** Maysa A. Mobasher, Eman Ibrahim Ahmed, Nora Y. Hakami, Mousa O. Germoush, Nabil S Awad, Dina M. Khodeer

**Affiliations:** 1 Department of Pathology, Biochemistry Division, College of Medicine, Jouf University, Sakaka 72388, Saudi Arabia; 2 Pharmacology and Therapeutics Department, College of Medicine, Jouf University, Sakaka 72346, Saudi Arabia; 3 Pharmacology Department, Faculty of Medicine, Fayoum University, Fayoum 63511, Egypt; 4 Department of Medical Laboratory Technology, Faculty of Applied Medical Sciences, King Abdulaziz University, Jeddah 21423, Saudi Arabia; 5 Biology Department, College of Science, Jouf University, Sakaka 72388, Saudi Arabia; 6 Department of Genetics, Faculty of Agriculture and Natural Resources, Aswan University, Aswan 81528, Egypt; 7 College of Biotechnology, Misr University for Science and Technology, Giza 12563, Egypt; 8 Department of Pharmacology & Toxicology, Faculty of Pharmacy, Suez Canal University, Ismailia 41522, Egypt

**Keywords:** *Glycyrrhiza glabra* extract, mesenchymal stem cells, cisplatin, rat, hepatotoxicity

## Abstract

Drug-induced liver damage is a life-threatening disorder, and one major form of it is the hepatotoxicity induced by the drug cisplatin. In folk medicine, Licorice (*Glycyrrhiza glabra* (is used for detoxification and is believed to be a potent antioxidant. Currently, the magically self-renewable potential of bone marrow mesenchymal stem cells (BM-MSCs) has prompted us to explore their hepatoregenerative capability. The impact of *G. glabra* extract (GGE) and BM-MSCs alone and, in combination, on protecting against hepatotoxicity was tested on cisplatin-induced liver injury in rats. Hepatic damage, as revealed by liver histopathology and increased levels of serum aspartate aminotransferase (AST), alanine aminotransferase (ALT), alkaline phosphatase (ALP), and malondialdehyde (MDA), was elevated in rats by received 7 mg/kg of cisplatin intraperitoneally. The combination of GGE and BM-MSCs returned the enzyme levels to near the normal range. It also improved levels of liver superoxide dismutase (SOD) and glutathione (GSH) and reduced MDA levels. Additionally, it was found that when GGE and BM-MSCs were used together, they significantly downregulated caspase9 (Casp9), nuclear factor kappa-light-chain-enhancer of activated B cells (NF-kB), and interleukin-1β (IL-1β), which are involved in severe proinflammatory and apoptotic signaling cascades in the liver. Moreover, combining GGE and BM-MSCs led to the normal result of hepatocytes in several examined liver histological sections. Therefore, our findings suggest that GGE may have protective effects against oxidative liver damage and the promising regenerative potential of BM-MSCs.

## 1. Introduction

Cis-Diamminedichloroplatinum (II) (cisplatin) is a common platinum-based anticancer medication [1]. Cisplatin has been used in treating 40% to 60% of patients suffering from cancers, for instance, testicular and colorectal tumors, and is considered the most promising drug for these patients [1]. The use of the chemotherapeutic drug cisplatin is associated with the development of acute renal damage (AKI) [2]. Cisplatin-induced kidney injury is predominantly attributable to proximal tubular damage from tubular uptake via the basolateral membrane transporter [3]. Tubulointerstitial damage is histopathological, but AKI only shows acute tubular necrosis [4]. AKI causes acute tubular necrosis, while cisplatin causes interstitial fibrosis [5]. Despite its potent anticancer activity, cisplatin is a double-edged sword because it can lead to serious toxicity in the liver. The liver is the body’s biggest solid organ in the body and is involved in most of the vital metabolic reactions. Cisplatin is characterized by its rapid diffusion into various tissues, especially the liver [6]. Even when cisplatin is administered at low doses, its long-term effect on the liver induces huge hepatotoxicity, including hepatic necrosis, liver cord breakdown, and localized inflammation [6].

Cisplatin-induced hepatotoxicity starts with the excessive generation of reactive oxygen species (ROS) and a concomitant increase in oxidative stress in the liver [7]. The massively generated ROS exhaust the hepatic antioxidant defense system, as is evident by the reduction of glutathione (GSH) levels, the exhaustion of antioxidant enzymes such as superoxide dismutase (SOD), and the increased levels of malondialdehyde (MDA) [8]. This effect is associated with the elevation of alanine transaminase (ALT), aspartate aminotransferase (AST), and alanine phosphatase (ALP) levels in the serum. These specific biomarkers are typically found in the hepatocyte’s cytoplasm and are released in the serum only after hepatocellular damage [9].

Furthermore, the accumulation of the intracellular ROS induces proinflammatory cytokines via the nuclear factor kappa-light-chain-enhancer of activated B cells (NF-kB) and the pro-apoptotic pathways by means of caspase 9 (Casp9) [10]. Additionally, many studies reported that cisplatin itself induced the overexpression of proinflammatory cytokines such as interleukin-1β (IL-1β) [11,12]. Consequently, cisplatin-induced serious histopathological alterations in the liver, including multifocal hepatocellular necrosis and mononuclear inflammatory cell infiltration. In addition, it increased mitotic activity in the liver, leading to dysplastic changes. Many studies have focused on relieving cisplatin-induced liver toxicity because cisplatin has therapeutic value as an anticancer drug [13,14]. Nature provides many herbal plants with beneficial therapeutic effects. One of the widely used herbal medicines is Licorice, that is extracted from the roots of the *Glycyrrhiza* species (Leguminosae family) [15]. In folk medicine, *Glycyrrhiza glabra* extract (GGE) has been an agent of choice in treating liver injury for many years [16].

A previous study reported that GGE contains valuable hepatoprotective flavonoids, which displayed a magical effect to reduce inflammation and apoptosis in the liver; this might explain the hepatoprotective effect of GGE [15]. Magnesium isoglycyrrhizinate (MgIG) is a stereoisomer of the licorice root’s glycyrrhizic acid, and it is the main compound that acts as an anti-inflammatory and hepatoprotective medication [15]. Also, glycyrrhetinic acid is hepatoprotective because it inhibits tumor necrosis factor-alpha (TNF-α) and caspase-3 (Casp3) [17]. In 2018, Sohail et al. studied the bioactive components of GGE and revealed that GGE contained various bioactive components with high antioxidant potential [18]. Many researchers studied the hepatoprotective potential of GGE [19]. These studies reported that GGE detoxified cisplatin-induced hepatic damage through the enhancement of cisplatin metabolism and the suppression of the massive release of ROS and their associated hepatic inflammation and apoptosis [7,15].

Recently, regenerative medicine has focused on using bone marrow mesenchymal stem cells (BM-MSCs) to enhance tissue engineering [20]. These self-renewable cells have revolutionized the field of tissue engineering with their ease of isolation, manipulability, and incredible regenerative potential [21]. Their abilities to proliferate, establish daughter cell lines, and repair injured tissues are of great interest to researchers [22]. Another advantage is their ability to dampen the inflammatory responses of various tissues [23]. The therapeutic effects of BM-MSCs are caused by many different stromal cells. This is because BM-MSCs can turn into functional hepatic cells and produce a series of growth factors and cytokines that can stop inflammatory responses, reduce hepatocyte death, reverse liver fibrosis, and improve hepatocyte function [24]. 

This study’s goal was to investigate the positive ameliorative role of BM-MSCs, GGE, and a combination of BM-MSCs and GGE in improving the hepatotoxic effect of cisplatin in rats. This can be achieved by biochemical, histological, and molecular studies.

## 2. Materials and Methods

### 2.1. Sample Collection and Preparation of Methanolic Extract from GGE Roots

The roots of GGE were collected from the online local market (https://www.cosmicelement.com/products/100-pure-cosmic-element-licorice-root-powder-pure-glycyrrhiza-glabra-mulethi-yashtimadhu-natural-halal-and-iso-certified-natural-expectorant-soothes-sore-throat-candy-flavoring-agent-superfood-8oz-227g) (accessed on 21 Novamber 2021). The plant root was ground to a coarse powder, and extractions were performed with methanol (95%) by the Soxhlet extraction procedure. In a rotary evaporator, the methanolic extracts were evaporated until they were dry and then concentrated under a vacuum at 40 to 50 °C. The extract was collected and stored in dark, airtight bottles until use [25]. 

### 2.2. Phytochemical Analysis and Antioxidant Activity of GGE

Gas chromatography–mass spectrometry (G.C.–M.S.) analysis, the total phenolic contents (TPC), total flavonoid contents (TFC), and ferric ion-reducing antioxidant power (FRAP) were estimated for GGE [26]. TPC was determined using the Folin–Ciocalteu reagent [27] and expressed in mg/g as the Gallic Acid Equivalent (GAE). The determination of flavonoids was carried out by the Aluminum Chloride Method [28]. TFC was measured in mg/g as the Quercetin Equivalent (Q.E.) [28]. FRAP assay was performed to estimate the antioxidant power of GGE [29], and the positive reference standard was ascorbic acid.

### 2.3. BM-MSCs Preparation

Rat BM-MSCs were received from the unit of Biochemistry, Faculty of Medicine Cairo University’s. The stem cells were isolated and manipulated as reported [30]. Briefly, the iliac crest was aspirated for bone marrow, and the samples were deposited in heparinized tubes. An ultrasonographic-guided intrasplenic injection was used to apply the bone marrow stromal cells.

### 2.4. Animal Model’s Design

**Figure 1:**
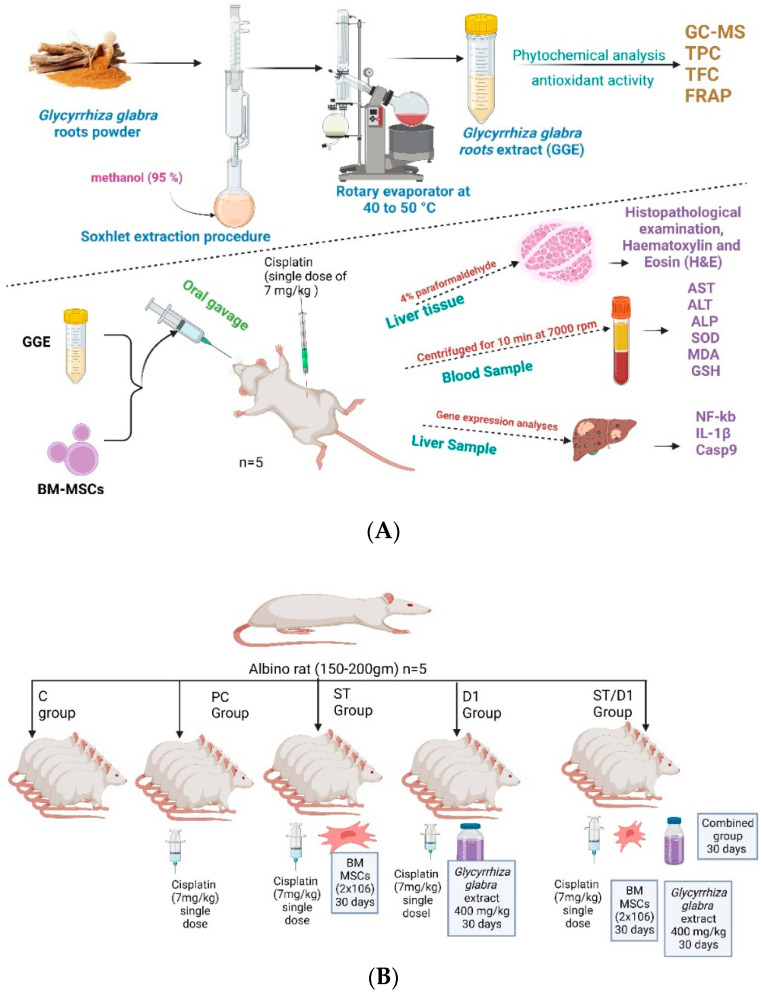
Experimental design and animal model.: (**A**) The experimental design. (**B**) The animal model with doses and duration.

Male albino rats (Sprague-Dawley) weighing 130 to 160 g each. The animals were housed in a room with a controlled air temperature (25 ± 2 °C) and maintained in a light: dark (12:12) cycle (Figure 1A). All animal experiments techniques were approved by the Local Committee of Bioethics (LCBE), Jouf University (No. 4-05-43). The rats were given an unrestricted supply of pellet food and unrestricted access to water. They were adapted for two days before we started the experiment. Healthy males were chosen at random and split into five groups, each with five rats (Figure 1B):

1. The control group got saline.
2. The cisplatin group (P.C. group): 7 mg/kg of cisplatin was injected intraperitoneally (I.P.) as a single dose to induce liver damage (Positive management).
3. The BM-MSCs group (Mesenchymal Stem Cells from Bone Marrow—St group): 7 mg/kg of cisplatin was injected intraperitoneally (I.P.) as a single dose, and on the following day, rats began receiving 2 × 10^6^ BM-MSCs per day in phosphate buffer solution (PBS) by intravenous (IV) injection for 1 month.
4. The GGE group (licorice-based drug D1 group): 7 mg/kg of cisplatin was injected intraperitoneally (I.P.) as a single dose, and the following day, rats received 400 mg/kg/day orally of GGE for one month.
5. The group consisting of BM-MSCs and GGE (Combinational treatment—St/D1 group) 7 mg/kg of cisplatin was injected intraperitoneally (I.P.) as a single dose, and the following day, rats received 2 × 10^6^ BM-MSCs in PBS by IV injection plus 400 mg/kg/day of GGE one month [31].

### 2.5. Blood and Liver Tissue Collection

At the end of the experiment, the animals were slaughtered, and blood was collected to evaluate biochemical parameters. After scarification, the liver tissues were rapidly removed, cleaned with physiological saline, and divided into two sections. The first section was placed in a Triazole reagent for the study of gene expression and kept at −20 °C. For histological analysis, the second piece of liver tissue was fixed in a 15% formaldehyde solution.

#### 2.5.1. Biochemical Markers of Serum

Collected blood samples were centrifuged for 10 min at 7000 rpm using a microcentrifuge to isolate the serum. The liver biomarkers (AST, ALT, and ALP) were estimated as reported [8]. The antioxidants and oxidative markers renal malondialdehyde (MDA), superoxidase dismutase (SOD), and glutathione (GSH) levels were measured as reported [32,33,34].

#### 2.5.2. Histopathological Examination, Hematoxylin, and Eosin (H&E)

Specimens that had previously been fixed in formalin were typically dehydrated in an increasing sequence of alcohols, cleaned in xylol, and then ultimately embedded with paraffin. Tissues with a thickness of between 4 and 5 μm were sectioned and prepared for H&E staining. The tissue slides were evaluated, and compared to the controls that corresponded to them, and finally, photographs were taken. Digital image capture equipment was utilized during the gathering of these photos (Olympus CX40; Olympus, Tokyo, Japan) [24].

### 2.6. Gene Expression Analyses 

Changes in the mRNA level of NF-kB, IL-1β, and Casp9 were measured. The total RNA was isolated from the liver tissues using the High Pure RNA Kit according to the manufacturer’s protocol (Roche, Germany). The RNA concentration and quality were determined spectrophotometrically at 260 nm and by the A260/A280 ratio, respectively. Subsequently, 0.1 μg of RNA from each sample was subjected to reverse transcription using the HyperScript™ First-strand synthesis kit (GeneAll, Seoul, Republic of Korea). A real-time polymerase chain reaction (RT-PCR) was performed with a Rotor-Gene Q (Qiagen) using RealQ Plus 2× Master Mix Green (Ampliqon, Odense, Denmark) [35]. The thermal cycling conditions involved an initial activation step for 15 min at 95 °C followed by 40 cycles, including a denaturation step for 20 s at 95 °C and a combined annealing/extension step for 60 s at 60 °C. Melting curves were analyzed to validate a single PCR product of each primer. The fold change in expression of each target mRNA relative to glyceraldehyde 3-phosphate dehydrogenase (GAPDH) was determined using the 2^–ΔΔct^ method. Primer sequences are stated in Table 1. 

### 2.7. Statistical Analysis 

Results are presented as means of 3 independent replicates. Analysis of Variance (one-way ANOVA test) with Tukey post hoc test was used to analyze the data; statistical analysis tests were done with SPSS version 21. Results were shown as mean ± S.D. and were considered significant at a *p*-value < 0.05 (Tukey post hoc test). Presented distinct lowercase letters denote statistically significant differences in comparison to the group serving as the control. *** *p* < 0.001, ** *p* < 0.01, n.s.: *p*-value > 0.05 is considered non-significant (*n* = 5).

## 3. Results

### 3.1. Phytochemical Analysis and the Antioxidant Power of GGE

**Figure 2:**
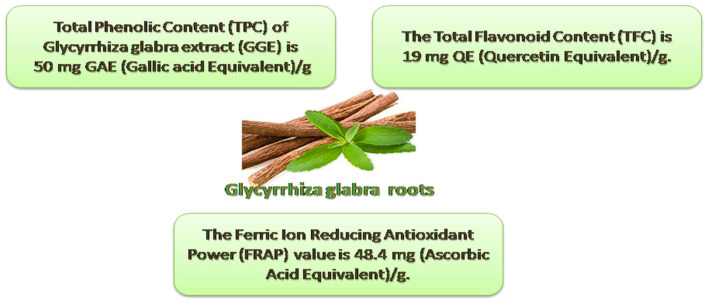
TPC, TFC, and FRAP in *Glycyrrhiza glabra* methanolic extract.

The results showed that the TPC of the GGE was 50 mg gallic acid equivalent/g. The TFC was 19 mg quercetin equivalent/g. The FRAP value was 48.4 mg of the ascorbic acid equivalent/g (Figure 2).

### 3.2. Biochemical Parameters

**Figure 3:**
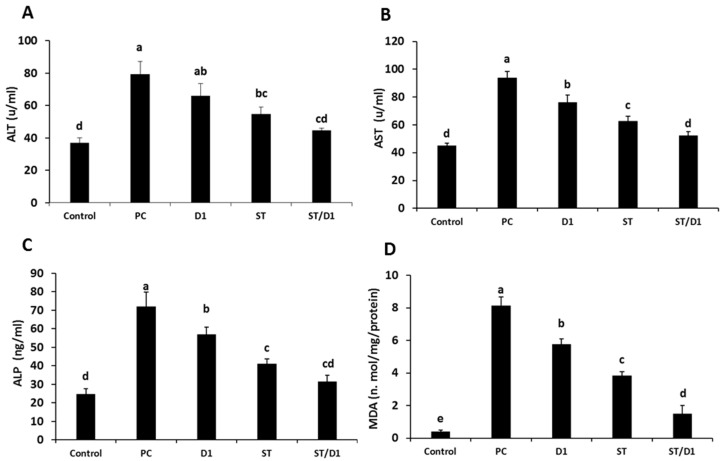
The effect of the treatment groups (Control, Cisplatin (P.C.), Cisplatin + GGE (D1), Cisplatin + BM-MSCs (St), Cisplatin + BM-MSCs + GGE (St/D1)) on the renal functions in rats, (**A**) ALT and (**B**) AST; (**C**) ALP and (**D**) MDA. Different alphabetic letter/s being significantly different (*p* < 0.05).

The methanolic extract of *G. glabra* roots and the BM-MSCs were studied for their hepatoprotective and antioxidant effects on liver damage from cisplatin in rats. Monitoring specific biomarker as ALT, AST, ALP, MDA, SOD, and GSH helped us to determine the extent of liver protection. The levels of liver biomarkers (ALT, AST, ALP), MDA, SOD, and GSH graphically represented the levels in test groups against the negative control group (Figure 3 and Table 2). Cisplatin treatment caused a considerable increase in liver biomarkers and MDA levels (Figure 3), associated with a considerable decline in the levels of SOD and GSH, compared with the control group (Table 2). The groups treated with BM-MSCs, GGE, or BM-MSCs plus GGE had an obvious increase in the levels of SOD and GSH allied with a decline in the levels of liver biomarkers, and MDA compared with the group given cisplatin, but left untreated. In the group receiving the combined BM-MSCs and GGE, levels of SOD and GSH were increased significantly, allied with a decline in the levels of liver biomarkers and MDA compared with the group that received cisplatin but was left untreated, the group that received GGE, and the group that was treated with BM-MSCs (Figure 3). Our findings might support the hepatoprotective potential of GGE combined with the influence exerted by BM-MSCs in the relief of oxidative liver injury.

### 3.3. Liver Histopathology 

**Figure 4:**
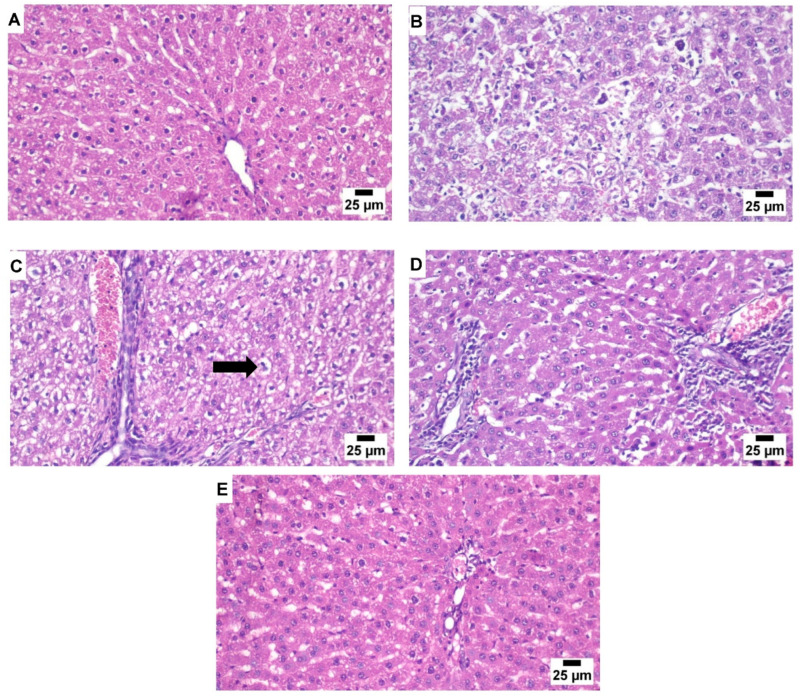
(**A**) The control group’s hepatic tissue has normal histological structure with radiating cords of hepatocytes surrounding the central vein. (**B**) Liver tissue from the Cisplatin group showing necrosis of the hepatic hepatocytes with evidence of dysplastic changes. (**C**) Liver tissue from the BM-MSCs group showing moderate vacuolated hepatocytes (arrow). (**D**) Liver tissue from the GGE group showing portal inflammation. (**E**) Liver tissue from the GGE and BM-MSCs combined group showing an apparently normal portal area with healthy adjacent hepatocytes (*n* = 5).

Hepatocytes in the control group’s livers appeared to be arranged in a normal lobular architecture, and the liver’s central veins and radiating hepatic cords were clearly visible under the microscope. Histological examination of the portal triads revealed typical architecture consisting of branched hepatic arteries, portal veins, and bile ducts. (Figure 4A). 

The administration of cisplatin resulted in serious histopathological alteration. Multifocal areas of hepatocellular necrosis were frequently detected in the affected individuals. The hepatic lobules showed numerous excessive vacuolated hepatocytes associated with the accumulation of eosinophilic, karyorrhectic debris and a variable number of mononuclear inflammatory cell infiltrations. Dysplastic changes were observed in a few sections associated with increased mitotic activity (Figure 4B). 

In the stem cell-treated group, the examined hepatic section showed apparently normal hepatocytes in several hepatic lobules. Fewer sections showed the multifocal aggregation of mononuclear inflammatory cells in the hepatic lobules as well as in the portal areas. The widening of the hepatic sinusoids revealed a marked dilation in some circumstances (Figure 4C). The GGE-treated group showed an increased pathological alteration compared to the BM-MSCs group. Multifocal inflammatory areas were noticed in the affected hepatic parenchyma that was characterized by a moderate number of mononuclear inflammatory cell aggregations. Some examined sections showed apparently normal hepatic tissue (Figure 4D). The GGE and BM-MSCs combined group revealed that hepatic parenchyma has significantly improved. The examination of the hepatic lobules showed apparently normal hepatocytes in several examined sections with intact portal areas and hepatic sinusoids (Figure 4E).

### 3.4. Gene Expression Analysis

**Figure 5:**
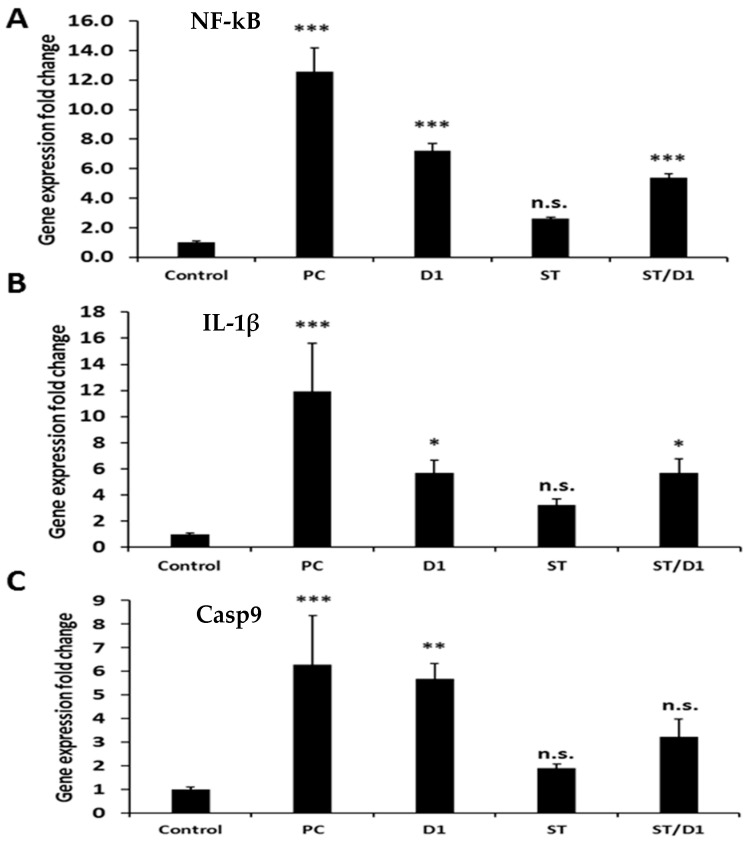
The effect of treated groups (Control, Cisplatin (P.C.), Cisplatin + GGE (D1), Cisplatin + BM-MSCs (St), Cisplatin + BM-MSCs + GGE (St/D1)) on gene expression profiling of the liver in rats. (**A**) NF-kB, (**B**) IL-1β, and (**C**) Casp9. *** highly significant *p* < 0.001, ** significant *p* < 0.005, * significant *p* < 0.05 and n.s mean non-significant.

The change in gene expression fold was monitored for NF-kB, IL-1β, and Casp9, which are involved in serious inflammatory and apoptotic signaling cascades in the liver (Figure 5). Normalizing agent, B-actin was utilized to validate the single polymerase chain reaction (PCR) product of each primer, and all data were the means of three replicates and presented as the mean plus or minus the standard deviation (S.D.). It was observed that NF-kB, IL-1β, and Casp9 were upregulated in the group given cisplatin but left untreated, compared with rats that served as the negative control. Rats treated with BM-MSCs, GGE, or BM-MSCs plus GGE, clearly showed that NF-kB, IL-1β, and Casp9 were downregulated compared with the group given cisplatin but left untreated. Interestingly, by monitoring how much each target mRNA gene’s expression changed (NF-kB, IL-1β, and Casp9) in the rats received the BM-MSCs and/or GGE, it was obvious that the NF-kB, IL-1β, and Casp9 were significantly further downregulated compared to the rat’s taken cisplatin, but left untreated, the group taken GGE, and the group given BM-MSCs. This might be attributed to the combined hepatoprotective effect of the GGE plus BM-MSCs.

## 4. Discussion

Hepatotoxicity is one of the many negative effects of cisplatin, an anticancer drug used to treat malignancies of solid organs [36,37]. Clinicians need to be aware of the anticipated consequences of its hepatotoxicity [38]. In the hospital context, recovery following an acute injury is essential to reduce patient morbidity and death [39]. In this investigation, we demonstrated that cisplatin was capable of inducing considerable hepatotoxicity in mice, as shown by increases in their blood-liver enzyme activity, upregulation of proinflammatory markers, and abnormalities in histopathology.

The small size of the cisplatin molecule lets it get through the cell membrane and affects the DNA structure in the nucleus. Although the exact mechanisms by which cisplatin causes liver damage to remain unknown, one possible explanation for such an effect could be that oxidative stress and apoptosis are likely contributors [40]. Other reports have found that cisplatin reduced glutathione as an antioxidant marker and, at the same time, raised lipid peroxidation as an oxidative stress marker [41]. 

When oxidative stress is present, impairment of the body’s antioxidant system may occur or ROS may be overproduced [42,43]. Therefore, in the present study, the group that received 7 mg/kg of cisplatin I.P., but was left untreated, displayed serious hepatic histopathological alterations, multifocal areas of hepatocellular necrosis, and a variable number of infiltrated mononuclear inflammatory cells [44]. Moreover, this group showed a significant elevation in ALT, AST, ALP, and MDA levels associated with a considerable decline in SOD and GSH levels compared with the negative control group given saline. 

Numerous hypotheses about cisplatin-induced hepatotoxicity have been published. These reports have discussed the drug’s pathways in the development of lipid peroxidation, mitochondrial dysfunction, DNA damage [7], and the destruction of structural proteins [40]. Free radicals and ROS are elevated as a result of the DNA damage caused by the binding of cisplatin’s platinum components to DNA. In our study, levels of NF-kB, IL-1β, and Casp9 were upregulated in the group given cisplatin but left untreated, compared with the negative control group. NF-kB and IL-1β are essential inducers of inflammatory cytokines in the liver [10]. Casp9 is a crucial regulator of the initiation of apoptosis and cytokine signaling in the liver [45,46] and belongs to the cysteine-aspartic protease (caspase) family. 

GGE and BM-MSCs have been widely utilized in the clinical treatment of cisplatin-induced hepatotoxicity that is directly related to the exhaustion of the hepatic antioxidant defense system [47]. The capacities of GGE or BM-MSCs to mitigate oxidative stress, reduce free radicals, and enhance the overall functioning of the liver have been published in prior research [48,49].

In this study, the examined hepatic sections of rats that had single cisplatin dosage and had been treated with GGE were characterized by a moderate number of mononuclear inflammatory cell aggregations compared with the group given cisplatin but left untreated. There was an obvious increase in the levels of SOD and GSH allied with low levels of ALT, AST, ALP, and MDA, associated with NF-kB, IL-1β, and Casp9 having been downregulated as well, compared with the group given cisplatin, but left untreated. A variety of pharmacologic effects, including anti-inflammatory, antioxidant, immunomodulatory, and hepatoprotection, have been attributed to licorice [50]. Our findings revealed that GGE was rich in considerable amounts of phenolic compounds and antioxidant flavonoids that could relieve hepatic oxidative stress [51]. Furthermore, owing to the molecular similarities between glycyrrhizic acid and adrenal cortex-secreted mineralocorticoid hormones and glycyrrhizic acid’s glucocorticoid action, Licorice has steroid-like anti-inflammatory effects comparable to those of hydrocortisone [52].

The mitigation effect may be due to three mechanisms of action. The first is the inhibition of hepatic apoptosis and necrosis. This inhibition takes place through the blockage of mitochondrial cytochrome C release [17], the suppression of TNF-α and Casp3 [53], and the inhibition of HMGB1 production by Kupffer cells [54]. The second effect occurs through anti-inflammatory and immune regulation [55]. This action is achieved by the suppression of the production of IL-6 and TNF-α produced by the lipid A moiety of lipopolysaccharides [56], as well as the inhibition of the synthesis of mitogen-activated protein kinase (MAPK), NF-kB, and IL-6 induced by paclitaxel [57]. The inhibition of the lytic pathway of the complementary system inhibits tissue damage from the membrane attack complex. The third effect occurs via the induction of liver enzyme activity by the triggering of the CYP3A pathway [58].

Recently, many studies have focused on the excellent regenerative potential of BM-MSCs and their capabilities of repairing injured tissues and dampening the inflammatory responses of various tissues [22,23]. BM-MSCs have paracrine therapeutic activities, mainly through trophic factors [59]. In regenerative medicine, BM-MSCs perform important therapeutic functions as secretors of growth factors, a small number of cytokines, and chemokines, among other soluble factors and trophic factors [60]. By controlling how much cytokines and other substances that cause inflammation are released, BM-MSCs create a hepatoprotective environment: they decrease inflammation, cellular death, and fibrosis while simultaneously promoting angiogenesis and tissue cell regeneration [61].

**Figure 6:**
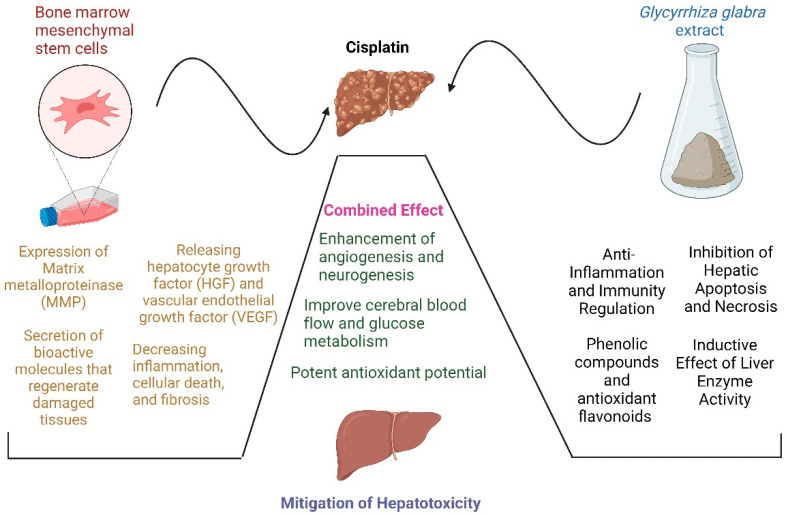
A summary of the suggested mechanism of *Glycyrrhiza glabra* and/or BM-MSCs on cisplatin-induced hepatocellular damage in rats.

We used an animal model in our experiment of cisplatin-induced hepatotoxicity that was treated with BM-MSCs, GGE, or BM-MSCs in combination with GGE. Extremely high levels of SOD and GSH was seen, and a decrease in ALT, AST, ALP, and MDA levels associated with the downregulation of NF-kB, IL-1β, and Casp9 in these groups compared with the group given cisplatin but left untreated. Consistent with previous research, our data showed that BM-MSCs were able to recover serum liver function markers dramatically and liver enzyme levels, consequently decreasing liver fibrosis in damaged rat livers [49]. The expression of matrix metalloproteinase (MMP) was upregulated by the BM-MSCs, whereas the expression of the tissue inhibitor of metalloproteinase (TIMP) was downregulated; these changes were mostly associated with fibrosis resolution and dramatically destroyed collagen fibers [62]. Two of the most well-studied trophic factors believed to be released by MSCs are hepatocyte growth factor (HGF) and vascular endothelial growth factor (VEGF) [63]. HGF is pivotal in the constructive control of hepatocyte proliferation [64]. One study reported that BM-MSCs were an essential component of the connective tissue that provides structural support for various functional cells [65]. Other researchers studied the therapeutic properties of BM-MSCs and their impact on regenerative medicine. It was found that BM-MSCs exerted their therapeutic effects through the secretion of bioactive molecules that regenerated damaged tissues [66] (Figure 6).

In the group treated with BM-MSCs plus GGE, our results revealed a considerable downregulation of Casp9, NF-kB, and IL-1β that might be attributed to the potent antioxidant potential of GGE and the complementary regenerative potential of the BM-MSCs and their ability to dampen various inflammatory responses. These results were strongly supported by the remarkable improvement of the hepatic parenchyma and the obvious amelioration of hepatocytes in several examined sections after treatment with the combined BM-MSCs and GGE. Our previous research showed that compared with either BM-MSCs alone or the drug silymarin alone, their combination was more effective [67]. The levels of liver enzymes in the serum were restored to those of the control group, suggesting that the combined therapies strongly augment the hepatic environment. As a consequence of this, the BM-MSCs were able to survive longer in the host tissue and were able to differentiate into hepatocytes more effectively, and the hepatic function was greatly improved. [49]. Interestingly, it was observed that the specific biomarkers, which are strongly related to oxidative liver injury, were normalized by the antioxidant potential of GGE combined with the regenerative potential of the BM-MSCs. Many studies stated that the combined synergistic effect of GGE and BM-MSCs could be because of the acceleration of BM-MSC differentiation and migration when BM-MSCs are combined with GGE [68,69]. Others have suggested that the combined effect reveals an enhancement of angiogenesis and neurogenesis [70,71]. Finally, they effectively regulate astrocytes and improve the flow of blood to the brain and the metabolism of glucose [72,73,74]. The actions of GGE, BM-MSCs, and the combined BM-MSCs and GGE are summarized in Figure 6.

## 5. Conclusions

In traditional medicine, GGE is used to treat liver dysfunction. *G. glabra* has a well-known hepatoprotective capability. Furthermore, BM-MSCs have extraordinary regenerative capabilities. In our research, we explored the hepatoprotective impact of GGE, BM-MSCs, and combined GGE and BM-MSCs on cisplatin-induced liver damage in rats. GGE combined with BM-MSCS significantly inhibited the elevated hepatic enzymes caused by cisplatin intoxication. This combination led to a remarkable improvement in the liver parenchyma and showed that hepatic function was improved and that blood liver enzyme levels were returned to those of healthy controls, demonstrating that the combined therapy worked synergistically to alter the hepatic environment.

## Figures and Tables

**Table 1:** Genes and primer sequences used for the real-time PCR.

Gene	Primers	Sequence (5′–3′)
NF-kB	Forward	GCAGCACTACTTCTTGACCACC
	Reverse	TCTGCTCCTGAGCATTGACGTC
IL-1β	Forward	CCACAGACCTTCCAGGAGAATG
	Reverse	GTGCAGTTCAGTGATCGTACAGG
Casp9	Forward	AGCCAGATGCTGTCCCATAC
	Reverse	CAGGAGACAAAACCTGGGAA
B-actin	Forward	GTGACATCCACACCCAGAGG
	Reverse	ACAGGATGTCAAAACTGCCC

**Table 2:** The effect of the treatment groups (Control, Cisplatin, Cisplatin + GGE, Cisplatin + BM-MSCs, and Cisplatin + BM-MSCs + GGE) on the SOD and GSH level in rats. Different alphabetic letter/s being significantly different (*p* < 0.05).

Caption	GSH (m. mol/mg/Protein)	SOD (u/mg/Protein)
Control	4.49 ± 0.43 ^a^	4.15 ± 0.50 ^a^
Cisplatin	3.55 ± 0.51 ^a,b^	0.47 ± 0.10 ^d^
GGE + Cisplatin	0.87 ± 0.19 ^d^	1.29 ± 0.31 ^c,d^
BM-MSCs + Cisplatin	2.51 ± 0.48 ^b,c^	2.15 ± 0.23 ^c^
BM-MSCs + GGE + Cisplatin	1.36 ± 0.44 ^c,d^	3.16 ± 0.23 ^b^
*p*-Value	<0.001	<0.001

## Data Availability

The data presented in this study are available in article.
